# Baker's Cyst

**DOI:** 10.7759/cureus.20403

**Published:** 2021-12-14

**Authors:** Arjun Nanduri, Thor S Stead, Graham E Kupsaw, Jason DeLeon, Latha Ganti

**Affiliations:** 1 Emergency Medicine, Lake Nona High School, Orlando, USA; 2 Medicine, The Warren Alpert Medical School of Brown University, Providence, USA; 3 Neuroscience, Brown University, Providence, USA; 4 Emergency Medicine, Lakeland Regional Health, Lakeland, USA; 5 Emergency Medicine, Envision Physician Services, Plantation, USA; 6 Emergency Medicine, University of Central Florida College of Medicine, Orlando, USA; 7 Emergency Medicine, Hospital Corporation of America (HCA) Healthcare Graduate Medical Education Consortium, Orlando, USA

**Keywords:** knee pain, emergency medicine, ultrasonography, popliteal cyst, baker's cyst

## Abstract

The authors present a case of a Baker's cyst in the right leg of an 86-year-old woman, whose presentation was more typical for a deep venous thrombosis. Both conditions have inflammation and acute calf pain. The clinical manifestations, imaging findings, and treatment of this common emergency department presentation are discussed.

## Introduction

A Baker's cyst, also known as a popliteal cyst, is caused by fluid distention of the gastrocnemio-semimembranosus bursa and results in a painful synovial-lined fluid sac outside the knee joint due to its communication with the knee cavity [1]. Most often it presents in adults aged 35-70 years. A popliteal cyst is associated with commonly found intra-articular knee disorders, such as osteoarthritis and meniscus tears [2]. Other underlying conditions can include Charcot joint [1], degenerative arthropathy [3], and post-traumatic injury in athletes [4]. Most patients with a popliteal cyst have no symptoms. When symptomatic, the most common complaint is a palpable swelling at the popliteal area with associated vague pain [5]. The incidence of Baker's cyst ranges from 5-38% [6]. In a population of 399 patients with knee pain, the prevalence of popliteal cysts was noted to be 25.8%, increasing in frequency with age [7]. The authors present a classic case of Baker's cyst on the degenerative knee of an elderly woman.

## Case presentation

The patient is a healthy 86-year-old lady with no medical problems, who does not take any medications. She presents to the emergency department with right knee pain and swelling. It does not hurt to touch it, but it is difficult to bend it. The patient explained she had been on a long car ride of 16 hours the previous day. She denies any fevers, chills, chest pain, shortness of breath, nausea, vomiting, diarrhea, abdominal pain, headache, or urinary symptoms. She has no history of blood clots and has never been on a blood thinner.

Her vitals are within normal limits, with temperature 98.3ºF, heart rate 85 beats per minute, blood pressure 147/75 mmHg, respiratory rate 16 breaths per minute, and oxygen saturation of 98% on room air.

Physical examination demonstrates swelling, reduced range of motion, and joint effusion of the right knee. There are no signs of erythema, and the skin is intact. There is no tenderness to palpation of the effusion. The right lower extremity is slightly warmer than the left, and attempting to bend the knee beyond 30 degrees results in pain. The calf itself is non-tender, and there are no palpable cords. The thigh area also is non-tender to palpation. The patient has normal femoral, popliteal, and dorsalis pedis pulses. There are no signs or symptoms of compartment syndrome in the right lower extremity. Any pain the patient has is in proportion to the edema, the peripheral circulation is intact, capillary refill is not delayed, and there is no numbness, tingling, or paresthesia. Her laboratory evaluation is unremarkable except for a mildly elevated c-reactive protein and is summarized in Table 1.

**Table 1:** Patient's laboratory values BUN: blood urea nitrogen; GFR: glomerular filtration rate

Lab	Reference range	Value
Sodium	135-145 mmol/L	131 L
Potassium	3.5-5.3 mmol/L	4.1
Chloride	98-107 mmol/L	104
Carbon Dioxide	21-32 mmol/L	24
Anion Gap		3
BUN	7-18 mg/dL	10
Creatinine	0.6-1.3 mg/dL	0.6
Estimated GFR	< 60	>60
Glucose	74-106 mg/dL	208 H
Calcium	8.4-10.2 mg/dL	9.2
C-Reactive Protein	0-0.300 mg/dL	7.12 H
White blood cell count	4.1-9.3 K/mm3	6.9
Hemoglobin	12.1-15.1 gm/dL	12.1
Hematocrit	35.5-46.9 %	35.9
Platelet Count	150-450 K/mm3	234
Erythrocyte sedimentation rate	0-20 mm/hr	12

**Figure 1:**
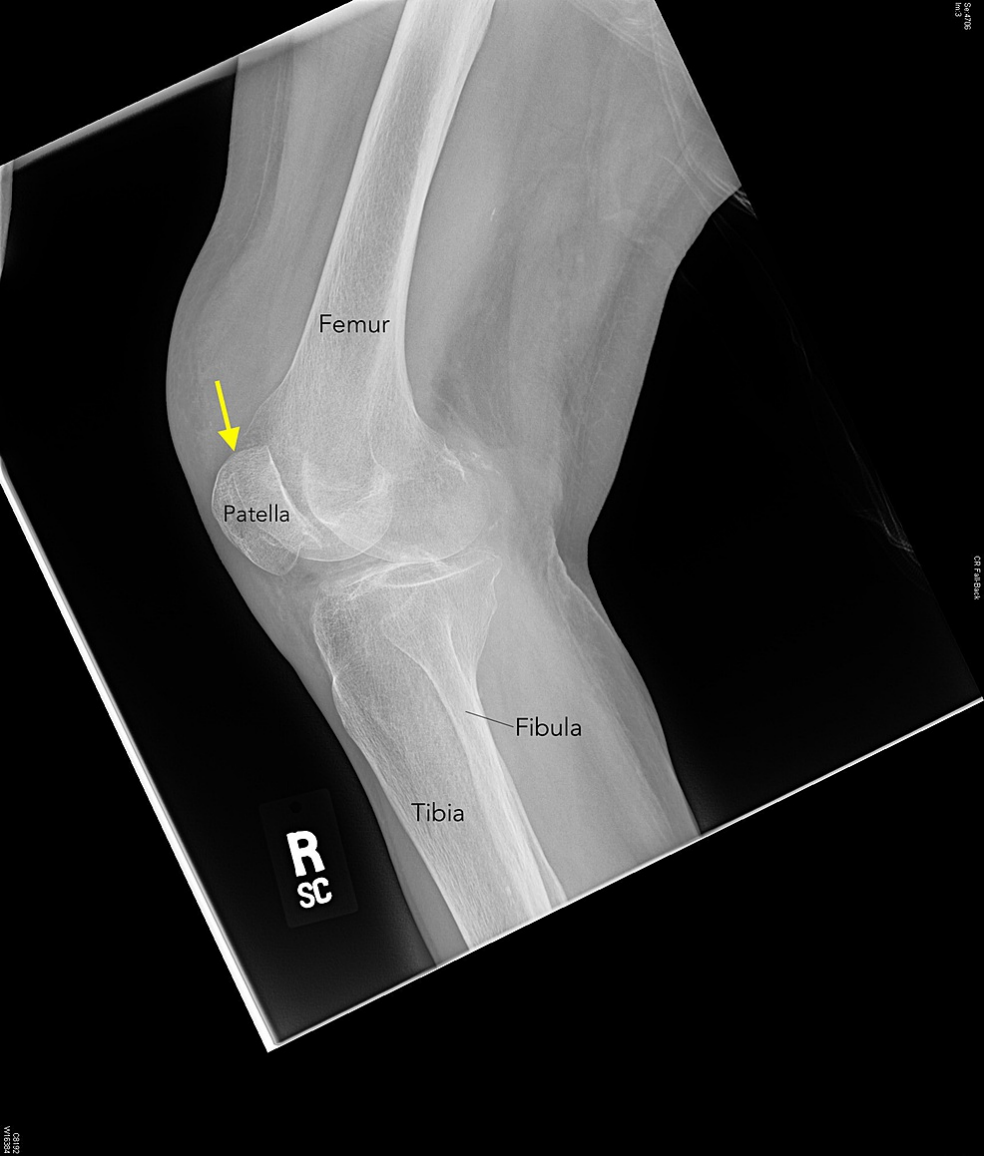
Knee radiograph demonstrating small joint effusion in the suprapatellar recess (arrow)

Radiograph of the right knee demonstrates mild osteoarthritis of the medial and lateral knee compartments of the right knee, and a small joint effusion in the suprapatellar recess (Figure 1).

**Figure 2:**
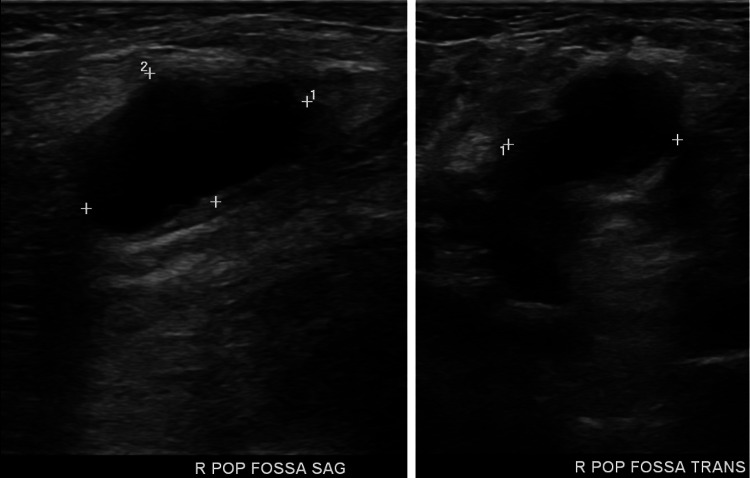
Ultrasonography of lower extremity reveals a popliteal cyst (crosshatches)

Ultrasonography of the right lower extremity reveals no deep vein thrombosis from the mid-calf to the common femoral vein. Observed is a popliteal cyst in sagittal and transverse views (Figure 2).

The patient was given Ketorolac in the emergency department and was discharged home with detailed information on her condition given to both her and her spouse, as well as follow-up with primary care.

## Discussion

The diagnosis of a popliteal cyst is most easily made via ultrasonography. Typical findings include anechoic or hypoechoic fluid between the semimembranosus and medial gastrocnemius tendons, and a posterior soft tissue mass or cyst, with an average size of 10cm3 [8]. Magnetic resonance imaging (MRI) will depict a Baker's cyst as a well-defined unilocular or multilocular cystic mass, located posteromedially, arising between the tendon of the semimembranosus and the medial head of gastrocnemius [9,10]. Fluid signal intensity is seen in all sequences in cases of popliteal cysts [10,11].

The most common differential diagnoses include deep vein thrombosis (DVT), cystic masses such as synovial or ganglion cysts, solid masses such as sarcoma and lymphoma, and popliteal artery aneurysms. Of these, DVT is the most common differential diagnosis, especially in the emergency department. In situations where ready imaging is not available, sometimes low molecular weight heparin is administered empirically for presumed DVT. If it is in fact a Baker's cyst rather than a DVT, heparin treatment can actually result in compartment syndrome [12].

**Figure 3:**
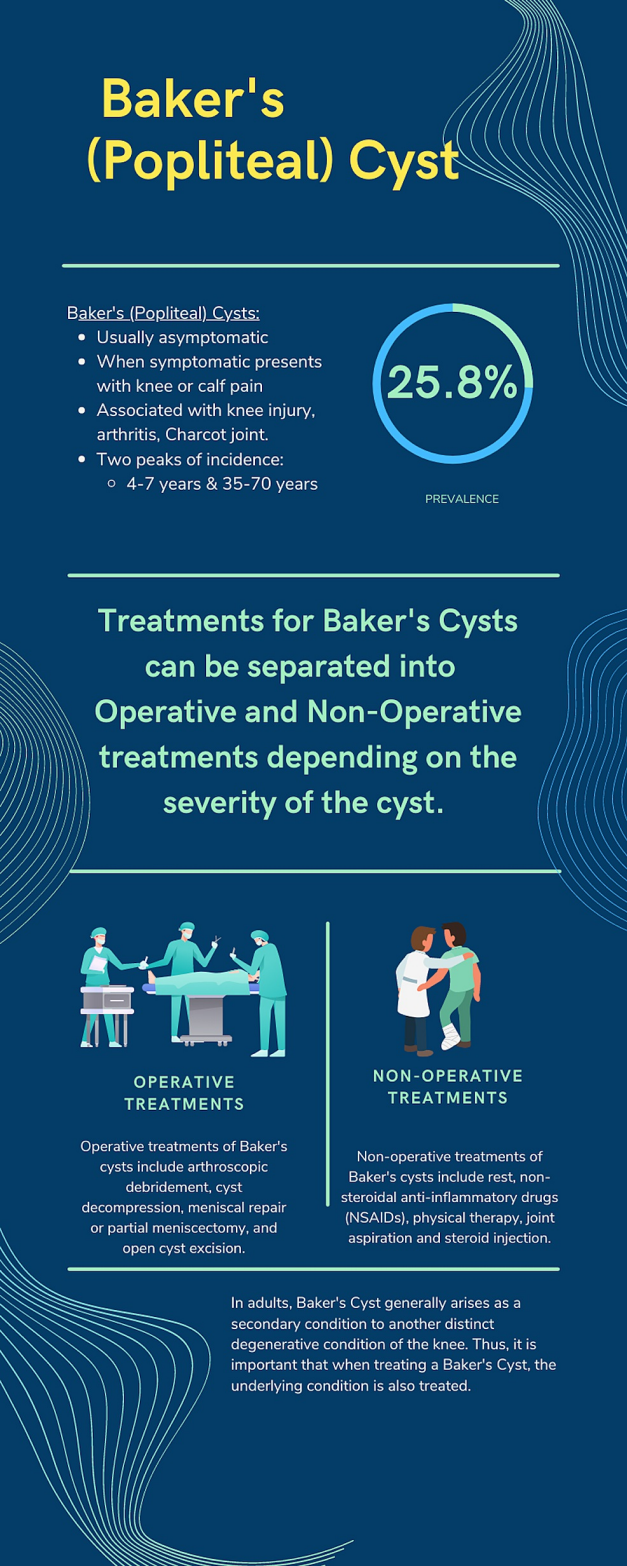
Infographic describing Baker's (popliteal) cyst Created by Arjun Nanduri on canva.com (Canva Pty Ltd, Surry Hills, Australia)

No treatment is necessary for asymptomatic Baker's cysts. Treatment for symptomatic Baker's cysts can be operative or non-operative (Figure 3) [13].

Initially, conservative non-operative management is preferred. Options include rest and activity restriction, oral or topical non-steroidal anti-inflammatory drugs (NSAIDs), physical therapy, and joint aspiration. Operative management includes arthroscopic debridement, cyst decompression, open cyst excision to surgically remove the cyst, and meniscal repair or partial meniscectomy. The latter often results in cyst recurrence [1]. An important detail to note is the existence of an underlying knee-joint disorder that caused the cyst. Successfully resolving the underlying disorder would reduce the amount of synovial fluid reaching the cyst, thus alleviating the condition. A systematic review of non-operative and operative treatment [13] found that that intracystic corticosteroid injection with cyst fenestration is an effective non-operative treatment method.

## Conclusions

A Baker's or popliteal cyst is a benign condition that often presents with acute knee or calf pain. The diagnosis is easily made with imaging and helps to distinguish it from a deep venous thrombosis. Acute management is generally conservative with rest, ice, elevation, and analgesics. Addressing the underlying condition can be curative. Occasionally, surgical intervention may be warranted and is typically performed arthroscopically.
